# Social Risk Factors and Disparities in Advanced Cardiovascular-Kidney-Metabolic Syndrome

**DOI:** 10.1001/jamanetworkopen.2026.10702

**Published:** 2026-05-05

**Authors:** Obinna Ekwunife, Xuemeng Wang, Raphael A. Fraser, Alexander Yunke, Jennifer A. Campbell, Rebekah J. Walker, Leonard E. Egede

**Affiliations:** 1 Division of Population Health, Department of Medicine, Jacobs School of Medicine and Biomedical Sciences, University at Buffalo, Buffalo, New York; 2 Jacobs School of Medicine and Biomedical Sciences, University at Buffalo, Buffalo, New York

## Abstract

**Importance:**

Social risk factors contribute to cardiovascular, kidney, and metabolic disease; however, their associations with advanced cardiovascular-kidney-metabolic (CKM) syndrome and variation across demographic subgroups remain unclear.

**Objective:**

To examine the association between individual social risk factors and advanced CKM syndrome and to assess whether these associations vary by age group, sex, and race and ethnicity.

**Design, Setting, and Participants:**

This is a cross-sectional study of adults aged 30 years or older from the National Health and Nutrition Examination Survey 2005 to 2018 cycles. Data analysis was conducted from July to October 2025. Analyses incorporated survey weights to generate nationally representative estimates.

**Exposures:**

Five social risk domains: economic instability, poor neighborhood environment, limited education, limited health care access, and poor social and/or community context.

**Main Outcomes and Measures:**

The primary outcome was advanced CKM syndrome (stages 3-4) defined using American Heart Association criteria. Following tests for interactions by age, race and ethnicity, and sex, weighted logistic regression tested associations between social risks and advanced CKM syndrome, stratified by race and ethnicity and sex.

**Results:**

Among 28 218 participants (representing 165.8 million US adults; mean [SD] age, 52.6 [14.2] years; 14 402 female participants [52.0%]), 12 614 (44.7%) had advanced CKM syndrome. No significant interactions were found for age. Economic instability was associated with higher odds of advanced CKM syndrome among non-Hispanic Black (odds ratio [OR], 1.27; 95% CI, 1.08-1.50) and non-Hispanic White adults (OR, 1.22; 95% CI, 1.08-1.37). Poor neighborhood environment was significant for non-Hispanic Black adults (OR, 1.20; 95% CI, 1.03-1.38). Limited education was associated with advanced CKM syndrome among non-Hispanic White adults (OR, 1.29; 95% CI, 1.13-1.48). Poor social and/or community context was associated across all groups, with the highest OR among Hispanic adults (OR, 1.72; 95% CI, 1.43-2.08). By sex, social risks were more associated with advanced CKM syndrome in women, with associations for men less prevalent and limited to economic instability, poor neighborhood environment, and poor social and/or community context.

**Conclusions and Relevance:**

In this cross-sectional study of US adults, multiple social risks factors were associated with advanced CKM syndrome, with meaningful variation by race and ethnicity and sex. Integrating social risk screening into CKM syndrome prevention and tailoring interventions to high-risk subgroups may help reduce disparities and slow CKM syndrome progression.

## Key Points

**Question:**

Are individual social risk factors associated with advanced cardiovascular-kidney-metabolic (CKM) syndrome, and do these associations vary by age group, sex, and race and ethnicity?

**Findings:**

In this cross-sectional study with 28 218 participants, factors associated with advanced CKM syndrome included economic instability among non-Hispanic Black and White adults, poor neighborhood environment among non-Hispanic Black adults, limited education among non-Hispanic White adults, and poor social and/or community context across all groups. All 4 social risk factors were associated with higher odds of advanced CKM syndrome among both men and women, although the associations were less prevalent among men.

**Meaning:**

These findings suggest that targeted screening and subgroup-specific interventions may reduce disparities and slow CKM syndrome progression.

## Teaser

This cross-sectional study investigates associations between individual social risk factors and advanced cardiovascular-kidney-metabolic (CKM) syndrome.

## Introduction

In the US, cardiovascular, kidney, and metabolic dysfunction are associated with excess morbidity, premature mortality, and substantial social and economic costs.^1,2,3,4,5,6,7,8^ Because the heart, kidneys, and metabolic systems are closely interconnected, dysfunction across these systems often cooccurs and has been defined by the American Heart Association (AHA) as cardiovascular-kidney-metabolic (CKM) syndrome.^9,10,11^ CKM syndrome severity is categorized into 5 stages on the basis of risk factors and established disease, ranging from stage 0 (no CKM syndrome risk factors) to stage 4 (clinical cardiovascular disease).^11^ Recent data indicate that up to 90% of US adults meet criteria for CKM syndrome, with 15% in advanced stages,^3,4^ and prevalence varying by sex and age.^12^ The burden is increasing over time and disproportionately affects marginalized populations.^13^

Beyond clinical risk factors, social conditions in which people live, work, and age also influence CKM syndrome development and outcomes.^14^ Adverse conditions, often described as social risks, are associated with poor CKM syndrome health.^11,15,16,17,18^ Social risk factors, such as unemployment, low income, food insecurity, and social isolation, have been associated with each component of CKM syndrome and contributing to disparities in morbidity and mortality.^13,19,20,21^ Individuals experiencing these social risks also have higher CKM syndrome prevalence^13^ and greater risk of premature mortality in advanced stages.^19^

The Healthy People 2030 framework outlines 5 domains of social determinants of health: financial circumstances, education, health care access, neighborhood environment, and social context.^22^ This framework has been applied to CKM syndrome, with cumulative social risk burden associated with a substantially higher likelihood of advanced disease.^20^ Although prior studies have documented disparities in CKM syndrome prevalence and linked cumulative social risk to CKM syndrome stages and mortality,^13,19,20^ less is known about how individual social risk domains are independently associated with advanced CKM syndrome within demographic subgroups. In particular, whether these associations differ by age, sex and race and ethnicity remains unclear. To address this gap, we examined the association between individual social risk factors and advanced CKM syndrome and assessed variations across age group, sex, and race and ethnicity.

## Methods

### Study Population

This cross-sectional study used data from the National Health and Nutrition Examination Survey (NHANES), a nationally representative survey designed to collect health data for US population.^23^ Ethics review for NHANES data collection was approved by the National Center for Health Statistics Research and as publicly available, deidentified data, review and informed consent were not required for this analysis. This study was conducted and reported in accordance with the Strengthening the Reporting of Observational Studies in Epidemiology (STROBE) reporting guideline for cross-sectional studies (see eMethods in Supplement 1).^24^ Recommended sampling weights were applied for the analyses.^25^ For this study, data from 2005 to 2018 cycles were included in the analytic dataset based on availability of data across the suite of social risk factors. Participants younger than 30 years were excluded because the AHA PREVENT equations used to define high 10-year cardiovascular risk (stage 3 CKM syndrome) are validated for adults aged 30 to 79 years; restricting the sample ensured appropriate risk estimation and consistent CKM syndrome stage classification. We further excluded participants with missing values for systolic blood pressure (SBP), total cholesterol, high-density lipoprotein, estimated glomerular filtration rate (eGFR),^26^ body mass index (BMI; calculated as weight in kilograms divided by height in meters squared), or hemoglobin A_1c_ (HbA_1c_), to ensure all individuals in the sample had sufficient data to calculate CKM syndrome stages. Data used to create the sample were obtained from the laboratory and examination datasets. The eGFR was calculated using the race-free version of eGFR equation.^26^

### CKM Syndrome Stages

CKM syndrome, categorized into stages 0 through 4, was created using the AHA statement,^11^ Aggarwal et al,^12^ and Zhu et al,^13^ to allow definition within the NHANES dataset. All variables were obtained from the demographic, examination, and laboratory datasets. Stage 0 (no CKM syndrome risk factors) was defined as participants with BMI less than 25 and waist circumference less than 88 cm for female participants and less than 102 cm for male participants who were not qualified for other stages. Stage 1 (excess or dysfunctional adiposity) was defined as participants with a BMI greater than or equal to 25 or increased waist circumference (female participants, ≥88 cm; male participants, ≥102 cm) or prediabetes. Prediabetes was defined as self-reported prediabetes or, if not self-reported diabetes, fasting blood glucose of 100 through 126 mg/dL (to convert to millimoles per liter, multiply by 0.0555) or HbA_1c_ of 5.7% through 6.5% (to convert to proportion of total hemoglobin, multiply by 0.01).

Stage 2 (metabolic risk factors and CKD) was defined as participants with at least 1 of the metabolic risk factors^1^: elevated fasting serum triglyceride levels greater than or equal to 135 mg/dL (to convert to millimoles per liter multiply by 0.0113); hypertension, defined as self-reported hypertension or, if not self-reported hypertension, SBP greater than or equal to 130 mm Hg or diastolic blood pressure greater than or equal to 80 mm Hg^3^; diabetes, defined as self-reported diabetes or, if not self-reported, HbA_1c_ greater than or equal to 6.5%;^4^ or metabolic syndrome, defined as greater than or equal to 3 of the following: elevated waist circumference, low high density lipoprotein level (males <40 mg/dL or females <50 mg/dL; to convert to millimoles per liter, multiply by 0.0259), fasting serum triglycerides greater than or equal to 150 mg/dL, elevated blood pressure (SBP ≥130 mm Hg, diastolic blood pressure ≥80 mm Hg and/or use of blood pressure-lowering medications), or prediabetes. Participants with moderate-to high-risk CKD according to the Kidney Disease: Improving Global Outcomes (KDIGO) criteria were also included in this stage.^27^ CKD stages were classified according to eGFR and urinary albumin-to-creatinine ratio.^26^

Stage 3 (subclinical CVD) was defined as participants with very-high-risk CKD by KDIGO criteria^28^ or high estimated 10-year CVD risk. The 10-year CVD risk was estimated using the AHA PREVENT equations.^29,30^ The PREVENT equations were developed by select members of the AHA Cardiovascular-Kidney-Metabolic Scientific Advisory Group. The risk equations were derived and validated in a large, diverse sample of over 6 million individuals.^29,30^ The information derived from the use of PREVENT is based on version 1.0.0. Subsequent updates and future adaptations of the software may yield different results and conclusions. High CVD risk was calculated as greater than or equal to 20% 10-year CVD risk. The PREVENT equations were applied for adults aged 30 to 79 years. To maximize data while providing conservative estimates, adults 80 years or older were assigned an age of 79. Variables with the following ranges were applied for PREVENT equation: total cholesterol 130 to 320 mg/dL (to convert to millimoles per liter multiply by 0.0259) , high-density lipoprotein 20 to 100 mg/dL, SBP 90 to 200 mm Hg, and eGFR 15 to 140 mL/min/1.73 m^2^, and BMI 18.5 to 39.9. For individuals whose variable values outside the specific ranges, values were assigned to nearest allowable upper or lower bound, respectively. Stage 4 (clinical CVD) was defined as participants who self-reported CVD, based on responding yes to whether a health care professional had ever diagnosed them with coronary heart disease, angina, heart attack, heart failure, or stroke.

CKM syndrome stages were then dichotomized into 2 levels: nonadvanced (stages 0–2) and advanced (stages 3–4). The outcome was coded as 0 for nonadvanced stages and 1 for advanced stages, such that the models estimate the odds of being in an advanced stage of the syndrome.

### Social Risk Factors

Social risk factors were defined using the social determinants of health framework, adapted from the modified Kaiser Family Foundation conceptual model,^18,31^ and aligned with the key domains outlined in the Healthy People 2030.^22^ Proxy indicators were chosen for each of the 5 social risk factor domains. Economic stability was defined using family poverty-to-income ratio and categorized as above 130% of federal poverty level vs 130% or less of poverty level. Neighborhood or Built Environment was operationalized as household food insecurity, assessed in NHANES using the US Department of Agriculture Food Security Survey Module, which includes validated questions regarding concerns about food sufficiency and affordability over the prior 12 months. On the basis of the standardized scoring, low and very low food security were classified as food insecurity (risk), while full and marginal food security were classified as food security (no risk). Education access was defined as lack of education and classified as high school and greater than high school or less than high school (12 grades). Health care access was defined as lack of health insurance and was classified as having insurance or not having insurance (at risk). Social or community context was assessed using depressive symptoms, as a proxy for social and emotional support challenges, measured with 9 questions from the Patient Health Questionnaire-9.^18,32,33^ The total score was calculated based on summing all questions and categorized into no depression (0-4) or depression.^5,6,7,8,9,10,11,12,13,14,15,16,17,18,19,20,21,22,23,25,26,27,28^ Each social risk was created as a dichotomous variable with no risk coded as 0 and risk in the domain coded as 1.

### Covariates

Demographic factors included age (30-49, 50-64, or ≥65 years), sex (female or male), race and ethnicity (Hispanic, non-Hispanic Black, non-Hispanic White, or other [ie, Asian, multiracial, or any other race and ethnicity not otherwise specified]), and marital status (married vs not married [widowed, divorced, separated, never married, or living with partner]). Race and ethnicity were self-reported by participants during the NHANES interview using standardized survey categories defined by the National Center for Health Statistics. Lifestyle factors included smoking status (never, former, or current), BMI (continuous), and diet defined using daily energy intake from the dietary interview—total nutrient intakes (day 1) and categorized as good (1600-3000 kcal/day) or poor (<1600 or >3000 kcal/day). Comorbidity factors included self-reported cancer, chronic obstructive pulmonary disease (COPD), and asthma, each added as a separate covariate.

### Statistical Analysis

Baseline characteristics of participants were summarized across all variables, grouped by nonadvanced stages and advanced stages, using weighted percentages, mean (SD), and unweighted counts. Logistic regression models were then used to test the association between social risk factors and CKM syndrome. For each model, CKM syndrome nonadvanced vs advanced stages were used as the outcome. First, individual models were fitted for each of the 5 social risk factors with a single factor serving as the primary independent variable. Second, interaction models were tested that included each of the 5 social risk factors and demographic factors (age, sex, and race and ethnicity). A separate model was run for each social risk factor with interaction terms of the social risk by age, social risk by sex, and social risk by race and ethnicity added to each model. Statistically significant interactions (*P* < .05) guided stratified analyses.

Stratified models were conducted where interaction terms met criteria for statistically significance. Within each stratified models, all 5 social risk factors and covariates were included. All statistical tests were 2-sided, and *P* < .05 was used to determine statistical significance. All analyses incorporated NHANES examination sampling weights and accounted for the complex, multistage, stratified sampling design (including strata and primary sampling units) using survey design procedures with Taylor series linearization for variance estimation. Statistical software R version 4.4.1 (R Project for Statistical Computing) was used in the analysis.

## Results

Table 1 shows the study population’s characteristics. Among 28 218 participants (representing 165.8 million US adults; mean [SD] age, 52.6 [14.2] years; 14 402 female participants [52.0%]), 6991 identified as Hispanic (12.7%), 5737 as non-Hispanic Black (10.1%), 12 435 as non-Hispanic White (69.8%), and 3055 as other (7.4%). Overall, 15 604 (55.3%) had nonadvanced CKM syndrome (stages 0-2) and 12 614 (44.7%) had advanced CKM syndrome (stages 3–4).

**Table 1.** Weighted Sample Characteristics for Adults, 2005 to 2018

Characteristic	Participants, No. (%)		
	Overall (N = 28 218, unweighted; N = 165 803 036 weighted;)	Nonadvanced CKM syndrome (stages 0-2) ( n = 15 604, unweighted; N = 99 451 495 weighted)	Advanced CKM syndrome (stages 3 or 4) (n = 12 614, unweighted; N = 66 351 540 weighted)
Age, mean (SD), y	52.6 (14.2)	48.1 (12.7)	59.4 (13.6)
Age category, y			
30-49	11 445 (45.2)	8794 (58.5)	2651 (25.4)
50-64	8724 (32.4)	4402 (29.4)	4322 (36.7)
≥65	8049 (22.4)	2408 (12.1)	5641 (37.9)
Sex			
Female	14 402 (52.0)	8053 (52.5)	6349 (51.1)
Male	13 816 (48.0)	7551 (47.5)	6265 (48.9)
Race and ethnicity			
Hispanic	6991 (12.7)	4398 (14.9)	2593 (9.5)
Non-Hispanic Black	5737 (10.1)	2571 (8.3)	3166 (12.6)
Non-Hispanic White	12 435 (69.8)	6660 (68.8)	5775 (71.4)
Other^a^	3055 (7.4)	1975 (8.0)	1080 (6.5)
Marital status			
Not married	11 999 (37.4)	6192 (35.2)	5807 (40.8)
Married	16 204 (62.6)	9404 (64.8)	6800 (59.2)
Smoking			
Never smoker	15 051 (53.4)	8872 (56.7)	6179 (48.6)
Former smoker	7606 (27.6)	3508 (24.0)	4098 (33.1)
Current smoker	5544 (18.9)	3208 (19.4)	2336 (18.3)
Body mass index, mean (SD)^b^	29.4 (6.7)	28.2 (6.1)	31.1 (7.2)
Diet Quality Proxy			
Bad (<1600 or >3000 kcal/d)	13 055 (46.4)	6948 (45.6)	6107 (47.6)
Good (1600-3000 kcal/d)	13 681 (53.6)	7829 (54.4)	5852 (52.4)
Economic stability, % of federal poverty level			
>130	18 073 (81.3)	10 255 (82.7)	7818 (79.2)
≤130	7679 (18.7)	4002 (17.3)	3677 (20.8)
Neighborhood or built environment			
Food security	22 554 (86.6)	12 560 (87.4)	9994 (85.6)
Food insecurity	5235 (13.4)	2797 (12.6)	2438 (14.4)
Education access			
High school and greater than high school	20 906 (83.7)	11 854 (85.3)	9052 (81.3)
Less than high school (12 grades)	7283 (16.3)	3737 (14.7)	3546 (18.7)
Health care access			
Insurance	22 931 (85.3)	11 891 (82.3)	11 040 (89.8)
No insurance (at risk)	5261 (14.7)	3699 (17.7)	1562 (10.2)
Social or community context			
No depressive symptom	19 804 (77.3)	11 429 (80.5)	8375 (72.5)
Depressive symptom	6429 (22.7)	2985 (19.5)	3444 (27.5)
Cancer			
No	25 124 (88.2)	14 488 (91.6)	10 636 (83.0)
Yes	3068 (11.8)	1102 (8.4)	1966 (17.0)
Chronic obstructive pulmonary disease			
No	26 013 (92.2)	14 797 (94.5)	11 216 (88.8)
Yes	2201 (7.8)	804 (5.5)	1397 (11.2)
Asthma			
No	24 395 (86.2)	13 838 (88.0)	10 557 (83.6)
Yes	3800 (13.8)	1754 (12.0)	2046 (16.4)

Abbreviation: CKM, cardiovascular-kidney-metabolic.

^a^ Other includes participants who identified as Asian, multiracial, or another race and ethnicity not classified as Hispanic, non-Hispanic Black, or non-Hispanic White, consistent with National Health and Nutrition Examination Survey categorization.

^b^ Body mass index is calculated as weight in kilograms divided by height in meters squared.

Significant interactions were observed for sex and race and ethnicity across multiple social risk domains; therefore, stratified models were conducted (eTable in Supplement 1). No significant interactions were observed for age. The results of the stratified logistic regression models examining the odds of advanced CKM syndrome (stages 3-4 vs 0-2) by race and ethnicity and sex are presented in Tables 2 and 3, respectively. In fully adjusted models, several social risk factors were significantly associated with increased odds of advanced CKM syndrome, with notable variation across subgroups. Economic instability was significantly associated with higher odds of advanced CKM syndrome among non-Hispanic Black adults (OR, 1.27; 95% CI, 1.08-1.50) and non-Hispanic White adults (OR, 1.22; 95% CI, 1.08-1.37), but not among Hispanic adults (OR, 1.12; 95% CI, 0.95-1.32) or those in the other race and ethnicity category (OR, 1.38; 95% CI, 0.98-1.94). By sex, economic instability was significantly associated with higher odds of advanced CKM syndrome in both female (OR, 1.23; 95% CI, 1.09-1.40) and male (OR, 1.21; 95% CI, 1.01-1.45) participants.

**Table 2.** Weighted Stratified Logistic Regression Model Examining the Association Between Social Risk Factors and Advanced CKM Syndrome by Race and Ethnicity^a^

Variable	OR (95% CI)							
	Non-Hispanic White		Non-Hispanic Black		Other		Hispanic	
	Unadjusted (individual)	Fully adjusted	Unadjusted (individual)	Fully adjusted	Unadjusted (individual)	Fully adjusted	Unadjusted (individual)	Fully adjusted
Economic stability, % of federal poverty level (reference, >130%)								
≤130%	1.39 (1.26-1.54)^b^	1.22 (1.08-1.37)^c^	1.35 (1.17-1.56)^b^	1.27 (1.08-1.50)^c^	1.78 (1.36-2.35)^b^	1.38 (0.98-1.94)	1.07 (0.92-1.24)	1.12 (0.95-1.32)
Neighborhood or built environment (reference, food security)								
Food insecurity	1.22 (1.05-1.42)^d^	1.21 (1.00-1.47)	1.24 (1.10-1.41)^b^	1.20 (1.03-1.38)^d^	1.73 (1.33-2.26)^b^	1.55 (1.04-2.32)^d^	1.12 (0.95-1.33)	1.12 (0.92-1.38)
Education access (reference, high school and greater than high school)								
Less than high school (12 grades)	1.78 (1.59-2.00)^b^	1.29 (1.13-1.48)^b^	1.60 (1.41-1.83)^b^	1.18 (0.98-1.42)	1.61 (1.23-2.11)^b^	1.12 (0.71-1.75)	1.07 (0.94-1.21)	0.93 (0.80-1.07)
Health care access (reference, yes insurance)								
No insurance (at risk)	0.60 (0.52-0.69)^b^	0.75 (0.64-0.89)^c^	0.53 (0.45-0.62)^b^	0.70 (0.57-0.85)^b^	0.66 (0.46-0.93)^d^	0.55 (0.33-0.92)^d^	0.44 (0.39-0.51)^b^	0.66 (0.56-0.79)^b^
Social or community context (reference, no depressive symptom)								
Depressive symptom	1.49 (1.35-1.65)^b^	1.42 (1.25-1.61)^b^	1.62 (1.39-1.88)^b^	1.40 (1.18-1.65)^b^	1.92 (1.51-2.43)^b^	1.61 (1.20-2.15)^c^	1.87 (1.64-2.13)^b^	1.72 (1.43-2.08)^b^
Age category, y (reference, 30-49 y)								
50-64	NA	2.86 (2.48-3.30)^b^	NA	3.13 (2.64-3.71)^b^	NA	4.08 (2.92-5.68)^b^	NA	3.54 (2.99-4.20)^b^
≥65	NA	7.80 (6.82-8.92)^b^	NA	7.74 (6.48-9.26)^b^	NA	10.21 (7.04-14.81)^b^	NA	8.23 (6.54-10.36)^b^
Sex (reference, female)								
Male	NA	1.32 (1.20-1.46)^b^	NA	1.02 (0.89-1.17)	NA	1.22 (0.85-1.75)	NA	1.28 (1.08-1.50)^c^
Marital status (reference, not married)								
Married	NA	0.91 (0.82-1.01)	NA	1.11 (0.95-1.31)	NA	1.37 (0.94-1.98)	NA	0.90 (0.78-1.04)
Smoking (reference, never smoker)								
Former smoker	NA	1.19 (1.08-1.31)^b^	NA	1.02 (0.83-1.24)	NA	0.79 (0.53-1.17)	NA	1.21 (1.01-1.46)^d^
Current smoker	NA	1.26 (1.08-1.46)^c^	NA	1.07 (0.90-1.27)	NA	1.29 (0.85-1.95)	NA	1.11 (0.92-1.35)
Body mass index^e^	NA	1.09 (1.08-1.09)^b^	NA	1.07 (1.06-1.08)^b^	NA	1.10 (1.07-1.13)^b^	NA	1.07 (1.06-1.08)^b^
Diet quality proxy (reference, bad)								
Good	NA	0.93 (0.83-1.04)	NA	1.00 (0.89-1.13)	NA	0.92 (0.69-1.23)	NA	0.98 (0.82-1.16)
Cancer (reference, no)								
Yes	NA	1.29 (1.13-1.48)^b^	NA	1.97 (1.45-2.66)^b^	NA	1.54 (0.84-2.85)	NA	1.39 (1.03-1.89)^d^
Chronic obstructive pulmonary disease (reference, no)								
Yes	NA	1.18 (0.95-1.46)	NA	1.88 (1.40-2.53)^b^	NA	1.37 (0.71-2.65)	NA	1.57 (1.06-2.32)^d^
Asthma (reference, no)								
Yes	NA	1.19 (1.03-1.37)^d^	NA	1.37 (1.12-1.67)^c^	NA	1.49 (0.91-2.44)	NA	1.32 (1.02-1.70)^d^

Abbreviations: CKM, cardiovascular-kidney-metabolic; NA, not applicable; OR, odds ratio.

^a^ The dependent variable is advanced CKM syndrome (stages 3-4). Nonadvanced stages (stages 0-2) were coded as 0, and advanced stages (stages 3 or 4) represent reference groups.

^b^ *P* < .001.

^c^ *P* < .01.

^d^ *P* < .05.

^e^ Body mass index is calculated as weight in kilograms divided by height in meters squared.

**Table 3.** Weighted Stratified Logistic Regression Model by Sex^a^

Variable	OR (95% CI)			
	Female		Male	
	Unadjusted (individual)	Fully adjusted	Unadjusted (individual)	Fully adjusted
Economic stability, % of federal poverty level (reference, >130%)				
≤130%	1.50 (1.36-1.64)^b^	1.23 (1.09-1.40)^c^	1.03 (0.93-1.14)	1.18 (1.03-1.36)^d^
Neighborhood or built environment (reference, food security)				
Food insecurity	1.32 (1.16-1.50)^b^	1.24 (1.05-1.47)^d^	1.01 (0.89-1.15)	1.21 (1.01-1.45)^d^
Education access (reference, high school and greater than high school)				
Less than high school (12 grades)	1.64 (1.48-1.81)^b^	1.32 (1.13-1.53)^b^	1.08 (0.97-1.20)	1.05 (0.91-1.22)
Health care access (reference, yes insurance)				
No insurance (at risk)	0.59 (0.52-0.67)^b^	0.72 (0.62-0.84)^b^	0.47 (0.40-0.54)^b^	0.67 (0.56-0.80)^b^
Social or community context (reference, no depressive symptom)				
Depressive symptom	1.73 (1.56-1.93)^b^	1.51 (1.34-1.70)^b^	1.40 (1.23-1.58)^b^	1.41 (1.19-1.68)^b^
Age category, y (reference, 30-49 y)				
50-64	NA	3.72 (3.18-4.35)^b^	NA	2.52 (2.20-2.88)^b^
≥65	NA	9.94 (8.44-11.71)^b^	NA	6.34 (5.37-7.49)^b^
Race and ethnicity (reference, Non-Hispanic White)				
Hispanic	NA	0.76 (0.66-0.88)^b^	NA	0.72 (0.61-0.85)^b^
Non-Hispanic Black	NA	1.87 (1.63-2.16)^b^	NA	1.44 (1.27-1.62)^b^
Other	NA	1.30 (1.01-1.68)^d^	NA	1.10 (0.90-1.35)
Marital status (reference, not married)				
Married	NA	0.96 (0.86-1.07)	NA	0.94 (0.83-1.06)
Smoking (reference, never smoker)				
Former smoker	NA	1.13 (0.98-1.30)	NA	1.23 (1.10-1.38)^b^
Current smoker	NA	1.31 (1.13-1.52)^b^	NA	1.23 (1.04-1.45)^d^
Body mass index^e^	NA	1.08 (1.07-1.08)^b^	NA	1.09 (1.08-1.11)^b^
Diet quality proxy (reference, bad)				
Good	NA	0.90 (0.80-1.01)	NA	1.00 (0.89-1.14)
Cancer (reference, no)				
Yes	NA	1.26 (1.08-1.46)^c^	NA	1.40 (1.18-1.67)^b^
Chronic obstructive pulmonary diseases (reference, no)				
Yes	NA	1.29 (1.04-1.60)^d^	NA	1.23 (0.93-1.65)
Asthma (reference, no)				
Yes	NA	1.31 (1.11-1.56)^c^	NA	1.16 (0.98-1.38)

Abbreviations: NA, not applicable; OR, odds ratio.

^a^ The dependent variable is advanced CKM syndrome (stages 3-4). Nonadvanced stages (stages 0-2) were coded as 0, and advanced stages (stages 3 or 4) represent reference groups.

^b^ *P* < .001.

^c^ *P* < .01

^d^ *P* < .05.

^e^ Body mass index is calculated as weight in kilograms divided by height in meters squared.

Poor neighborhood or built environment remained significantly associated with increased odds of advanced CKM syndrome in non-Hispanic Black adults (OR, 1.20; 95% CI, 1.03-1.38) and individuals of other race (OR, 1.55; 95% CI, 1.04-2.32), while associations for non-Hispanic White and Hispanic adults were not statistically significant. By sex, poor neighborhood or built environment was significantly associated with higher odds of advanced CKM syndrome in both female (OR, 1.24; 95% CI, 1.05-1.47) and male (OR, 1.21; 95% CI, 1.01-1.45) participants.

Limited education was significantly associated with higher odds of advanced CKM syndrome among non-Hispanic White adults (OR, 1.29; 95% CI, 1.13-1.48), but not other racial and/or ethnic groups. In the sex-stratified analyses, the association was observed in female participants (OR, 1.32; 95% CI, 1.13-1.53) but not in male participants.

Limited health care access was associated with lower odds of advanced CKM syndrome among non-Hispanic Black adults (OR, 0.70; 95% CI, 0.57-0.85), Hispanic adults (OR.0.66; 95% CI, 0.56-0.79), non-Hispanic White adults (OR, 0.75; 95% CI, 0.64-0.89), and in individuals of other race (OR, 0.55; 95% CI, 0.33-0.92). In sex-stratified analyses, this protective association was evident in female participants (OR, 0.72; 95% CI, 0.62-0.84), but not in male participants.

Across all racial and/or ethnic groups and both sexes, poor social or community context was consistently associated with greater odds of advanced CKM syndrome. The odds were highest among Hispanic adults (OR, 1.72; 95% CI, 1.43-2.08) and individuals of other race (OR, 1.61; 95% CI, 1.20-2.15), with elevated risks also observed among non-Hispanic White (OR, 1.42; 95% CI, 1.25-1.61) and non-Hispanic Black adults (OR, 1.40; 95% CI, 1.18-1.65). Similar patterns were present in female participants (OR, 1.51; 95% CI, 1.34-1.70) and male participants (OR, 1.41; 95% CI, 1.19-1.68).

## Discussion

In this large, nationally representative cross-sectional study of US adults, social risk factors were significantly associated with advanced stages of CKM syndrome, with associations varying by race and ethnicity and sex. Economic instability was most associated with advanced CKM syndrome among non-Hispanic Black and White adults, poor neighborhood environment among non-Hispanic Black adults, limited education among non-Hispanic White adults, and poor social and/or community context across all groups, particularly Hispanic adults and those from other racial and ethnic groups. By sex, all 4 risk factors were associated with higher odds of advanced CKM syndrome in women, whereas in men the odds were lower and limited to economic instability, poor neighborhood environment, and poor social or community context. Our findings contribute to the literature by demonstrating that multiple social risks differ across demographic subgroups, highlighting the need for tailored prevention strategies.

Our findings align with and extend prior research on the role of social risks factors in CKM syndrome. While prior studies by Zhu et al^13,19^ and Li et al^20^ examined the prevalence of CKM syndrome stages and cumulative social risk burden, our study differs in several important ways. First, we examined individual social risk domains simultaneously rather than cumulative indices. Second, we formally tested interaction terms and conducted fully adjusted stratified analyses by race and ethnicity and sex to identify subgroup-specific associations. Third, we focused specifically on advanced CKM syndrome (stages 3 and 4) as the outcome, rather than overall prevalence or mortality alone. These distinctions provide more granular, clinically actionable insight into which social risks are most associated with advanced disease within specific subgroups.

Our findings highlight the clinical importance of incorporating routine social risk screening into the management of patients at risk for CKM syndrome progression. Identifying factors such as economic instability, poor neighborhood environment, limited education, and weak social or community support may help clinicians better stratify risk and connect patients to appropriate resources, including community health workers, social services, or behavioral health support. The unexpected negative association between limited health care access and advanced CKM syndrome likely reflects selection and detection biases rather than a true negative association. In our sample, individuals without health insurance were typically younger and had a lower prevalence of diagnosed comorbidities such as cancer, COPD, and asthma. Because these individuals interact less frequently with the health system, they are less likely to have underlying conditions detected or classified as advanced CKM syndrome.^34^ This pattern may also reflect well-described phenomena such as the so-called healthy worker effect or healthy immigrant effect,^35,36^ in which relatively healthier individuals are overrepresented among uninsured populations. Consequently, the observed association likely reflects underdiagnosis and measurement limitations rather than a true reduction in CKM syndrome risk among those with limited health care access.

From a research and policy perspective, this study demonstrates that the influence of social risks on advanced CKM syndrome varies by race and ethnicity and sex. This subgroup-specific evidence supports developing more targeted, equity-focused interventions that move beyond one-size-fits-all approaches and integrates social determinants of health into CKM syndrome prevention and treatment. For example, medical-financial navigation, including linkage to food or housing assistance may be particularly impactful for non-Hispanic Black and White adults, given the association between economic instability and advanced CKM syndrome. Place-based strategies to improve neighborhood environments, such as mobile health units or community gardens, may better serve non-Hispanic Black and other race populations. Because women were more likely than men to have social risk factors associated with advanced CKM syndrome, interventions tailored to their needs may be especially beneficial. Integrating social risk screening and CKM syndrome care referral into obstetric, gynecological, and postpartum care, along with childcare, transportation support, and women-focused peer groups, could improve engagement. Delivering these services through settings frequently accessed by women, such as the Special Supplemental Nutrition Program for Women, Infants, and Children clinics or community health centers, may further enhance reach and effectiveness.

### Limitations

This study has several limitations. First, the cross-sectional design prevents establishing causality between social risks and advanced CKM syndrome. Second, key variables such as health insurance access and comorbidities were self-reported and may be affected by recall or reporting bias. Third, residual confounding may persist due to unmeasured factors like neighborhood environments or health care quality. Fourth, subgroup analyses may have limited statistical power. Fifth, because the data are cross-sectional, the direction of associations cannot be determined. Advanced CKM syndrome may influence social conditions such as health insurance access or depressive symptoms. Longitudinal studies are needed to clarify temporal relationships.

## Conclusions

This cross-sectional study found that multiple social risk factors, including economic instability, poor neighborhood environment, limited education, and poor social and/or community context, were significantly associated with advanced CKM syndrome, with notable differences by race and ethnicity as well as sex. These findings highlight the need for social risk screening and targeted interventions to reduce CKM syndrome disparities.

## Notes

Supplement 1. eMethods. Detailed interaction model outputs eTable. Interaction model: social risk factors

Supplement 2. Data Sharing Statement

## References

[1] Waters H, Graf M. America’s obesity crisis: the health and economic costs of excess weight. Milken Institute. 2018. Accessed September 3, 2025. https://milkeninstitute.org/sites/default/files/reports-pdf/Mi-Americas-Obesity-Crisis-WEB.pdf

[2] Whitlock G, Lewington S, Sherliker P, ; Prospective Studies Collaboration. Body-mass index and cause-specific mortality in 900 000 adults: collaborative analyses of 57 prospective studies. Lancet. 2009;373(9669):1083-1096. doi:10.1016/S0140-6736(09)60318-4 19299006 PMC2662372

[3] Turin TC, Tonelli M, Manns BJ, Ravani P, Ahmed SB, Hemmelgarn BR. Chronic kidney disease and life expectancy. Nephrol Dial Transplant. 2012;27(8):3182-3186. doi:10.1093/ndt/gfs052 22442392

[4] Salehidoost R, Mansouri A, Amini M, Aminorroaya Yamini S, Aminorroaya A. Diabetes and all-cause mortality, a 18-year follow-up study. Sci Rep. 2020;10(1):3183. doi:10.1038/s41598-020-60142-y 32081921 PMC7035261

[5] Chen F, Shi Y, Yu M, . Joint effect of BMI and metabolic status on mortality among adults: a population-based longitudinal study in United States. Sci Rep. 2024;14(1):2775. doi:10.1038/s41598-024-53229-3 38307987 PMC10837108

[6] Ostrominski JW, Arnold SV, Butler J, . Prevalence and overlap of cardiac, renal, and metabolic conditions in US adults, 1999-2020. JAMA Cardiol. 2023;8(11):1050-1060. doi:10.1001/jamacardio.2023.3241 37755728 PMC10535010

[7] Kim JE, Joo J, Kuku KO, . Prevalence, disparities, and mortality of cardiovascular-kidney-metabolic syndrome in US adults, 2011-2018. Am J Med. 2025;138(6):970-979.e7. doi:10.1016/j.amjmed.2025.01.031 39909293 PMC12092198

[8] Ahmad FB, Anderson RN. The leading causes of death in the US for 2020. JAMA. 2021;325(18):1829-1830. doi:10.1001/jama.2021.5469 33787821 PMC8145781

[9] Rangaswami J, Bhalla V, Blair JEA, .; American Heart Association Council on the Kidney in Cardiovascular Disease and Council on Clinical Cardiology. Cardiorenal syndrome: classification, pathophysiology, diagnosis, and treatment strategies: a scientific statement from the American Heart Association. Circulation. 2019;139(16):e840-e878. doi:10.1161/CIR.0000000000000664 30852913

[10] Koenen M, Hill MA, Cohen P, Sowers JR. Obesity, adipose tissue and vascular dysfunction. Circ Res. 2021;128(7):951-968. doi:10.1161/CIRCRESAHA.121.318093 33793327 PMC8026272

[11] Ndumele CE, Rangaswami J, Chow SL, ; American Heart Association. Cardiovascular-kidney-metabolic health: a presidential advisory from the American Heart Association. Circulation. 2023;148(20):1606-1635. doi:10.1161/CIR.0000000000001184 37807924

[12] Aggarwal R, Ostrominski JW, Vaduganathan M. Prevalence of cardiovascular-kidney-metabolic syndrome stages in US adults, 2011-2020. JAMA. 2024;331(21):1858-1860. doi:10.1001/jama.2024.6892 38717747 PMC11079779

[13] Zhu R, Wang R, He J, . Prevalence of cardiovascular-kidney-metabolic syndrome stages by social determinants of health. JAMA Netw Open. 2024;7(11):e2445309. doi:10.1001/jamanetworkopen.2024.45309 39556396 PMC11574692

[14] Alderwick H, Gottlieb LM. Meanings and misunderstandings: a social determinants of health lexicon for health care systems. Milbank Q. 2019;97(2):407-419. doi:10.1111/1468-0009.12390 31069864 PMC6554506

[15] Jilani MH, Javed Z, Yahya T, . Social determinants of health and cardiovascular disease: current state and future directions towards healthcare equity. Curr Atheroscler Rep. 2021;23(9):55. doi:10.1007/s11883-021-00949-w 34308497

[16] Chang R, Philip J, Javed U, . Unfavorable social determinants of health and risk of mortality in adults with diabetes: findings from the National Health Interview Survey. BMJ Open Diabetes Res Care. 2024;12(1):e003710. doi:10.1136/bmjdrc-2023-003710 38290988 PMC10828867

[17] Ozieh MN, Garacci E, Walker RJ, Palatnik A, Egede LE. The cumulative impact of social determinants of health factors on mortality in adults with diabetes and chronic kidney disease. BMC Nephrol. 2021;22(1):76. doi:10.1186/s12882-021-02277-2 33639878 PMC7916298

[18] Corwin T, Ozieh MN, Garacci E, Walker RJ, Egede LE. Association of social risk domains with poor cardiovascular risk factor control in US adults with diabetes, from 2006 to 2016. JAMA Netw Open. 2022;5(9):e2230853. doi:10.1001/jamanetworkopen.2022.30853 36083585 PMC9463604

[19] Zhu R, Wang R, He J, . Associations of cardiovascular-kidney-metabolic syndrome stages with premature mortality and the role of social determinants of health. J Nutr Health Aging. 2025;29(4):100504. doi:10.1016/j.jnha.2025.100504 39952015 PMC12180020

[20] Li J, Lei L, Wang W, . Social risk profile and cardiovascular-kidney-metabolic syndrome in US adults. J Am Heart Assoc. 2024;13(16):e034996. doi:10.1161/JAHA.124.034996 39136302 PMC11963957

[21] Yang Q, Shi P, Pan L, Huang Z. Social determinants of health (SDOH) associated with the risk of all-cause mortality and life expectancy in US adults with cardiovascular-kidney-metabolic syndrome: a NHANES 2001-2018 cohort study. BMC Cardiovasc Disord. 2025;25(1):369. doi:10.1186/s12872-025-04812-7 40375145 PMC12079812

[22] US Department of Health and Human Services. Healthy people 2030: social determinants of health. 2025. Accessed June 9, 2025. http://odphp.health.gov

[23] US Centers for Disease Control and Prevention: National Center for Health Statistics. National Health and Nutrition Examination Survey. 2025. Accessed March 19, 2026. https://www.cdc.gov/nchs/nhanes/index.html

[24] von Elm E, Altman DG, Egger M, Pocock SJ, Gøtzsche PC, Vandenbroucke JP; STROBE Initiative. The Strengthening the Reporting of Observational Studies in Epidemiology (STROBE) statement: guidelines for reporting observational studies. Lancet. 2007;370(9596):1453-1457. doi:10.1016/S0140-6736(07)61602-X 18064739

[25] US Centers for Disease Control and Prevention.National health and nutrition examination survey: weighting. 2025. Accessed March 19, 2026. https://wwwn.cdc.gov/nchs/nhanes/tutorials/Weighting.aspx

[26] Inker LA, Eneanya ND, Coresh J, ; Chronic Kidney Disease Epidemiology Collaboration. New creatinine- and cystatin c-based equations to estimate GFR without race. N Engl J Med. 2021;385(19):1737-1749. doi:10.1056/NEJMoa2102953 34554658 PMC8822996

[27] Kidney disease: improving global outcomes (KDIGO) CKD work group. KDIGO 2024 clinical practice guideline for the evaluation and management of chronic kidney disease. Kidney Int. 2024;105(suppl 4):S117-S314. doi:10.1016/j.kint.2023.10.018 38490803

[28] Stevens PE, Ahmed SB, Carrero JJ, ; Kidney Disease: Improving Global Outcomes (KDIGO) CKD Work Group. KDIGO 2024 clinical practice guideline for the evaluation and management of chronic kidney disease. Kidney Int. 2024;105(4S):S117-S314. doi:10.1016/j.kint.2023.10.018 38490803

[29] Khan SS, Coresh J, Pencina MJ, ; American Heart Association. Novel prediction equations for absolute risk assessment of total cardiovascular disease incorporating cardiovascular-kidney-metabolic health: a scientific statement from the american heart association. Circulation. 2023;148(24):1982-2004. doi:10.1161/CIR.0000000000001191 37947094

[30] Khan SS, Matsushita K, Sang Y, ; Chronic Kidney Disease Prognosis Consortium and the American Heart Association Cardiovascular-Kidney-Metabolic Science Advisory Group. Development and validation of the American Heart Association’s PREVENT equations. Circulation. 2024;149(6):430-449. doi:10.1161/CIRCULATIONAHA.123.067626 37947085 PMC10910659

[31] Artiga S, Hinton E. Beyond health care: the role of social determinants in promoting health and health equity. KFF. 2018. Accessed March 19, 2026. https://www.kff.org/racial-equity-and-health-policy/beyond-health-care-the-role-of-social-determinants-in-promoting-health-and-health-equity

[32] Kroenke K, Spitzer RL, Williams JB. The PHQ-9: validity of a brief depression severity measure. J Gen Intern Med. 2001;16(9):606-613. doi:10.1046/j.1525-1497.2001.016009606.x 11556941 PMC1495268

[33] Baah FO, Teitelman AM, Riegel B. Marginalization: conceptualizing patient vulnerabilities in the framework of social determinants of health—an integrative review. Nurs Inq. 2019;26(1):e12268. doi:10.1111/nin.12268 30488635 PMC6342665

[34] Centers for Medicare & Medicaid Services. Young adults and the Affordable Care Act: protecting young adults and eliminating burdens on families and businesses. 2024. Accessed March 19, 2026. https://www.cms.gov/cciio/resources/files/adult_child_fact_sheet

[35] Chowdhury R, Shah D, Payal AR. Healthy worker effect phenomenon: revisited with emphasis on statistical methods—a review. Indian J Occup Environ Med. 2017;21(1):2-8. doi:10.4103/ijoem.IJOEM_53_16 29391741 PMC5763838

[36] Elshahat S, Moffat T, Newbold KB. Understanding the healthy immigrant effect in the context of mental health challenges: a systematic critical review. J Immigr Minor Health. 2022;24(6):1564-1579. doi:10.1007/s10903-021-01313-5 34807354 PMC8606270

