# Supplementary material for: Social Risk Factors and Disparities in Advanced Cardiovascular-Kidney-Metabolic Syndrome
Source: JAMA Netw Open. 2026 May 5;9(5):e2610702. doi: 10.1001/jamanetworkopen.2026.10702 (PMC13147189; doi:10.1001/jamanetworkopen.2026.10702)
Supplement: Supplement 1. — eMethods. Detailed interaction model outputs eTable. Interaction model: social risk factors [file jamanetwopen-e2610702-s001.pdf]

Supplemental Online Content

Ekwunife O, Wang X, Fraser RA, et al. Social risk factors and disparities in advanced cardiovascular-kidney-metabolic syndrome. *JAMA Netw Open.* 2026;9(5):e2610702. doi:10.1001/jamanetworkopen.2026.10702

eMethods. Detailed interaction model outputs

eTable. Interaction model: social risk factors

This supplemental material has been provided by the authors to give readers additional information about their work.

© 2026 Ekwunife O et al. *JAMA Network Open*.

eMethods. Detailed interaction model outputs

For economic stability, age was not significant (p=0.316 for age 50-64 years old, p=0.58 for age >=65 years old), sex was significant (p <0.00), race/ethnicity was significant (p<0.01 for Hispanic group). For neighborhood or built environment, age was not significant (p=0.87 for age 50-64 years old, p=0.07 for age >=65 years old), sex was significant (p<0.00), race/ethnicity was significant (p<0.05 for other group). For education access, age was not significant (p = 0.1 for age 50-64 years old), sex was significant (p<0.001), race/ethnicity was significant (p<0.00 for Hispanic group). For health care access, age was not significant (p=0.92 for age 50-64 years old, p=0.58 for age >=65 years old), sex was significant (p<0.05), race/ethnicity was significant (p<0.01 for Hispanic group). For social or community context, age was not significant (p=0.23 for age 50-64 years old, p=0.54 for age >=65 years old), sex was significant (p<0.01), race/ethnicity was significant (p<0.01 for Hispanic group and p<0.05 for other group).

eTable. Interaction model: social risk factors

**Interaction model: social risk factors * age categories**	
	**OR (95% CI)**
130% and less of poverty level	1.38*** (1.21, 1.57)
50-64	2.90*** (2.61, 3.23)
> =65	7.44*** (6.64, 8.34)
130% and less of poverty level * 50-64	1.10 (0.91, 1.33)
130% and less of poverty level * > =65	0.95 (0.79, 1.14)
Food insecurity	1.48*** (1.31, 1.67)
50-64	2.90*** (2.64, 3.18)
> =65	7.48*** (6.76, 8.27)
Food insecurity * 50-64	1.16 (0.98, 1.37)
Food insecurity * > =65	1.24 (0.98, 1.56)
Less than high school/ 12 grades	1.08 (0.93, 1.26)
50-64	2.80*** (2.54, 3.08)
> =65	6.77*** (6.07, 7.56)
Less than high school/ 12 grades * 50-64	1.22 (0.96, 1.55)

© 2026 Ekwunife O et al. *JAMA Network Open*.

Less than high school/ 12 grades * > =65	1.34* (1.07, 1.68)
No insurance (at risk)	0.80*** (0.71, 0.91)
50-64	2.84*** (2.58, 3.12)
> =65	6.92*** (6.27, 7.63)
No insurance (at risk) * 50-64	1.01 (0.84, 1.21)
No insurance (at risk) * > =65	1.12 (0.75, 1.67)
Depression	1.85*** (1.63, 2.10)
50-64	2.96*** (2.65, 3.30)
> =65	7.37*** (6.65, 8.18)
Depression * 50-64	0.90 (0.76, 1.07)
**Interaction model: social risk factors * sex categories**	
	**OR (95% CI)**
130% and less of poverty level	1.50*** (1.36, 1.64)
male	1.17*** (1.08, 1.26)
130% and less of poverty level * male	0.69*** (0.60, 0.78)
Food insecurity	1.32*** (1.16, 1.50)
male	1.11** (1.03, 1.19)
Food insecurity * male	0.76*** (0.65, 0.89)
Less than high school/ 12 grades	1.64*** (1.48, 1.81)
male	1.14*** (1.06, 1.21)
Less than high school/ 12 grades * male	0.66*** (0.56, 0.76)
No insurance (at risk)	0.59*** (0.52, 0.67)
male	1.12** (1.04, 1.20)
No insurance (at risk) * male	0.79* (0.65, 0.96)
Depression	1.73*** (1.56, 1.93)
male	1.15*** (1.06, 1.24)
Depression * male	0.81** (0.68, 0.95)
**Interaction model: social risk factors * race/ethnicity categories**	
	**OR (95% CI)**
130% and less of poverty level	1.39*** (1.26, 1.54)
raceNon-Hispanic Black	1.39*** (1.26, 1.53)
raceOther	0.72*** (0.61, 0.84)
raceHispanic	0.63*** (0.56, 0.70)

© 2026 Ekwunife O et al. *JAMA Network Open*.

130% and less of poverty level * raceNon-Hispanic Black	0.97 (0.82, 1.16)
130% and less of poverty level * raceOther	1.29 (0.96, 1.71)
130% and less of poverty level * raceHispanic	0.77** (0.64, 0.93)
Food insecurity	1.22* (1.05, 1.42)
raceNon-Hispanic Black	1.42*** (1.31, 1.53)
raceOther	0.73*** (0.63, 0.84)
raceHispanic	0.60*** (0.54, 0.67)
Food insecurity * raceNon-Hispanic Black	1.02 (0.83, 1.25)
Food insecurity * raceOther	1.42* (1.06, 1.90)
Food insecurity * raceHispanic	0.92 (0.74, 1.14)
Less than high school/ 12 grades	1.78*** (1.59, 2.00)
raceNon-Hispanic Black	1.41*** (1.31, 1.51)
raceOther	0.77*** (0.67, 0.89)
raceHispanic	0.64*** (0.57, 0.71)
Less than high school/ 12 grades * raceNon-Hispanic Black	0.90 (0.76, 1.07)
Less than high school/ 12 grades * raceOther	0.90 (0.69, 1.19)
Less than high school/ 12 grades * raceHispanic	0.60*** (0.51, 0.70)
No insurance (at risk)	0.60*** (0.52, 0.69)
raceNon-Hispanic Black	1.57*** (1.45, 1.70)
raceOther	0.79** (0.68, 0.91)
raceHispanic	0.77*** (0.69, 0.85)
No insurance (at risk) * raceNon-Hispanic Black	0.89 (0.73, 1.09)
No insurance (at risk) * raceOther	1.10 (0.76, 1.61)
No insurance (at risk) * raceHispanic	0.75** (0.61, 0.90)
Depression	1.49*** (1.35, 1.65)
raceNon-Hispanic Black	1.41*** (1.30, 1.53)
raceOther	0.75*** (0.65, 0.86)
raceHispanic	0.56*** (0.51, 0.62)
Depression * raceNon-Hispanic Black	1.08 (0.91, 1.29)
Depression * raceOther	1.29* (1.01, 1.64)
Depression * raceHispanic	1.25** (1.07, 1.47)

© 2026 Ekwunife O et al. *JAMA Network Open*.
