# Supplementary material for: Social Risk Factors and Disparities in Advanced Cardiovascular-Kidney-Metabolic Syndrome
Source: JAMA Netw Open. 2026 May 5;9(5):e2610702. doi: 10.1001/jamanetworkopen.2026.10702 (PMC13147189; doi:10.1001/jamanetworkopen.2026.10702)
Supplement: Supplement 2. — Data Sharing Statement [file jamanetwopen-e2610702-s002.pdf]

Data Sharing Statement

Ekwunife. Social Risk Factors and Disparities in Advanced Cardiovascular-Kidney-Metabolic Syndrome. *JAMA Netw Open*. Published May 05, 2026.
doi:10.1001/jamanetworkopen.2026.10702

Data
Data available: No

Additional Information
Explanation for why data not available: Data Sharing Statement: This study used publicly available data from the Centers for Disease Control and Prevention (CDC) National Health and Nutrition Examination Survey (NHANES). The NHANES datasets are freely accessible through the CDC National Center for Health Statistics website at https://www.cdc.gov/nchs/nhanes/index.html.
